# Transcatheter edge-to-edge repair for post-surgical recurrent mitral regurgitation in hereditary spherocytosis: a case report

**DOI:** 10.1093/ehjcr/ytaf211

**Published:** 2025-04-29

**Authors:** Hiroto Yagasaki, Yukio Umeda, Takeki Suzuki, Ryota Watanabe, Toshiyuki Noda

**Affiliations:** Department of Cardiology, Gifu Prefectural General Medical Center, 4-6-1 Noisshiki, Gifu 500-8717, Japan; Department of Medicine, Indiana University School of Medicine, 340 West 10th Street, Fairbanks Hall, Suite 6200, Indianapolis, IN 46202-3082, USA; Department of Cardiovascular and Thoracic Surgery, Gifu Prefectural General Medical Center, 4-6-1 Noisshiki, Gifu 500-8717, Japan; Department of Cardiovascular and Thoracic Surgery, Gifu Prefectural General Medical Center, 4-6-1 Noisshiki, Gifu 500-8717, Japan; Department of Cardiology, Gifu Prefectural General Medical Center, 4-6-1 Noisshiki, Gifu 500-8717, Japan; Department of Cardiology, Gifu Prefectural General Medical Center, 4-6-1 Noisshiki, Gifu 500-8717, Japan

**Keywords:** Hereditary spherocytosis, Mitral regurgitation, Mitral valve transcatheter edge-to-edge repair, MitraClip, Case report

## Abstract

**Background:**

Management of mitral regurgitation (MR) in patients with hereditary spherocytosis (HS) poses unique challenges due to increased haemolysis risk. While surgical mitral valve repair is the standard treatment, the optimal strategy for recurrent MR after initial repair remains unclear, particularly regarding the safety and durability of transcatheter interventions in this high-risk population.

**Case summary:**

A 57-year-old woman with HS developed severe recurrent MR 4 years after initial surgical repair that intentionally omitted annuloplasty to minimize haemolysis risk. Given the risks of redo surgery and mechanical valve replacement, mitral valve transcatheter edge-to-edge repair (M-TEER) was performed. The procedure achieved successful MR reduction without causing haemolysis. At the 5-year follow-up, the patient maintained improved functional status with stable moderate MR and no evidence of haemolysis, despite her underlying condition.

**Discussion:**

This case demonstrates successful long-term outcomes of M-TEER for post-surgical recurrent MR in a patient with HS. The strategic approach—initial ring-less surgical repair followed by M-TEER—suggests a viable treatment pathway for patients with inherited haemolytic disorders, particularly when minimizing prosthetic material exposure is crucial.

**Learning points**

- Recognition of mitral valve transcatheter edge-to-edge repair as a viable treatment option for post-surgical recurrent mitral regurgitation in patients with hereditary haemolytic disorders.
- Understanding the clinical considerations in balancing durability and haemolysis risk when selecting valve interventions for patients with hereditary spherocytosis.

## Introduction

Hereditary spherocytosis (HS) is characterized by spherical erythrocytes that undergo premature splenic destruction.^1,2^ In cardiac valve interventions, mechanical haemolysis is a major concern for these patients.^3^ While surgical mitral valve repair (MVP) is the preferred treatment for mitral regurgitation (MR) due to lower haemolysis rates,^3,4^ the optimal strategy for recurrent MR in HS patients remains unclear. Mitral valve transcatheter edge-to-edge repair (M-TEER) has shown promise for recurrent MR after MVP,^5^ but experience in haemolytic disorders is limited. We report a case of M-TEER for post-surgical recurrent MR in a patient with HS during 5-year follow-up.

## Summary figure

**Figure:**
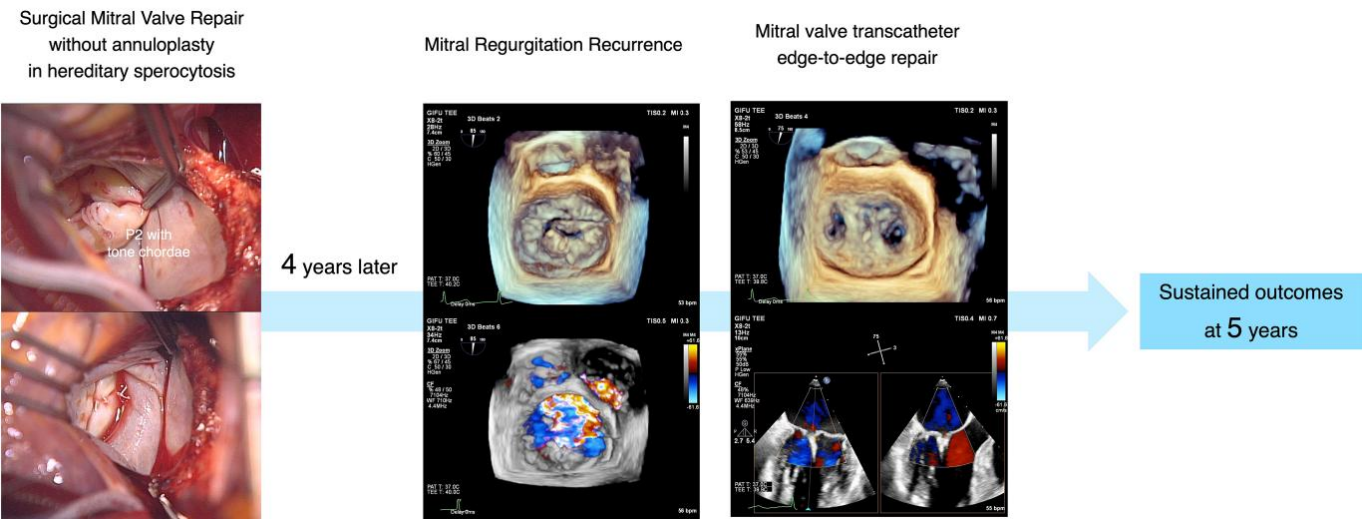


## Case presentation

A 57-year-old woman with HS and a history of surgical MVP presented to the hospital with exertional dyspnoea for 1 month. The patient was diagnosed with HS in her 30 s and had been managed conservatively without splenectomy. Four years before the current presentation, she underwent MVP for severe degenerative MR due to mid-portion of the posterior leaflet (P2) prolapse. She also had hypertension managed with olmesartan 20 mg once daily. The patient's vital signs showed blood pressure of 115/64 mmHg, heart rate of 66 beats per minute, and oxygen saturation of 99% on room air. Cardiovascular examination revealed a grade 3/6 holosystolic murmur, loudest at the apex, without extra heart sounds. Lungs were clear on auscultation. There was no jugular venous distention or lower extremity oedema. Physical examination and abdominal computed tomography showed no evidence of hepatosplenomegaly.

**Figure 1:**
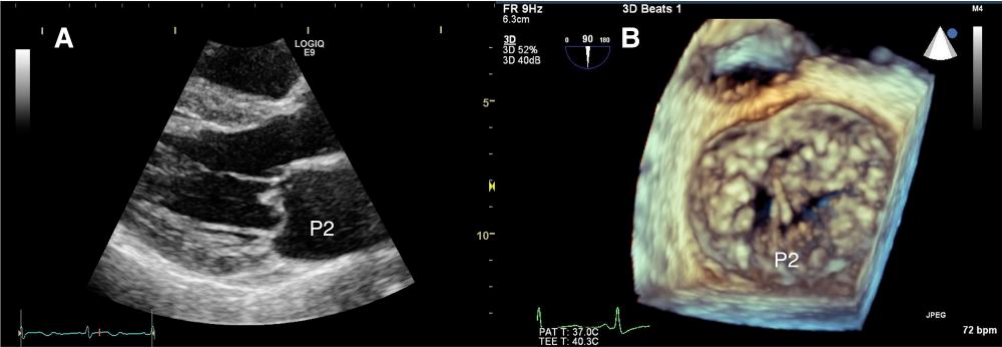
Preoperative echocardiography before initial surgical repair. (*A*) Parasternal long-axis view: P2 prolapse. (*B*) 3D transoesophageal echocardiography: P2 prolapse with chordal rupture.

**Figure 2:**
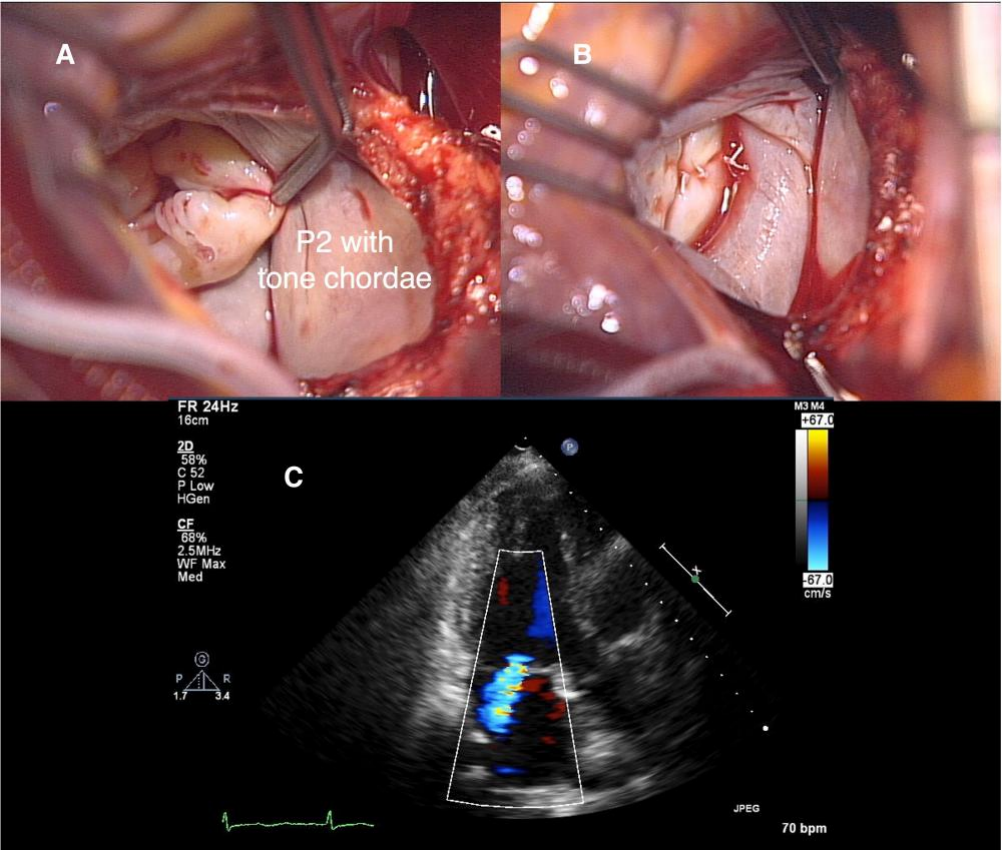
Initial surgical mitral valve repair. (*A*, *B*) Intraoperative images: P2 with ruptured chordae (*A*) and post-repair mitral valve (*B*). (*C*) Post-operative echocardiography: residual mild residual mitral regurgitation.

Preoperative echocardiography showed severe MR due to P2 prolapse with chordae tendineae rupture (*Figure 1A* and *B*, Supplementary material online, *Video S1*). The left atrium was mildly enlarged (diameter 42 mm) and left ventricular (LV) size was normal (end-diastolic diameter 49 mm) with preserved function (ejection fraction 75%). The initial surgery was performed using cardiopulmonary bypass. After reviewing surgical options, including an edge-to-edge repair technique (Alfieri stitch), the surgical team performed conventional P2 resection to minimize prosthetic material and sutures that could cause haemolysis. The surgery revealed a normal anterior leaflet with prolapsed and thickened P2 segment with torn chordae (*Figure 2A*). After triangular resection and suture of P2, a water test showed no obvious regurgitation (*Figure 2B*). Annuloplasty was intentionally omitted to avoid haemolysis. Post-operative echocardiography showed mild MR (*Figure 2C*, Supplementary material online, *Video S2*). During the operation, the patient received eight units of packed red blood cells and left the hospital on post-operative day (POD) 7 without major complications. However, on POD 22, she developed transient haemolytic anaemia, which resolved with appropriate transfusion (*Table 1*). This remained the only haemolytic episode requiring transfusion prior to her current presentation 4 years later.

**Table 1:** Haematological parameters at difference time points

	Pre-surgical MVP	Post-surgical MVP			Pre-M-TEER	Post-M-TEER			
Parameter (reference range)		7 POD (discharge)	22 POD	72 POD		1 month	1 year	3 years	5 years
Hb (11.6–14.8 g/dL)	10.1	10.5	6.2	11.2	10.8	11.2	11.2	11.6	11.4
Ht (35.1–44.4%)	28.6	30.0	17.1	31.4	31.5	32.5	31.9	33.2	33.1
MCV (83.6–98.2 fL)	87.7	88.8	86.1	87.5	92.6	90.8	90.1	90.9	90.2
MCHC (31.7–35.3 g/dL)	35.4	35.0	35.9	35.7	34.3	34.4	35.3	34.8	34.4
Reticulocyte count (0.8–22%)					3.9	8.5			
LDH (124–222 U/L)	234	214	840	220	215	228	185	189	221
T-bil (0.4–1.5 mg/dL)	2.08	1.51	3.53	1.91	1.40	1.42	1.52	1.41	1.86
D-bil (0–0.2 mg/dL)	0.82		0.74		0.13	0.12			
Hapt (30–200 mg/dL)	<10	<10	<10						

Hb, haemoglobin; Ht, haematocrit; MCV, mean corpuscular volume; MCHC, mean corpuscular haemoglobin concentration; LDH, lactate dehydrogenase; T-bil, total bilirubin; D-bil, direct bilirubin; Hapt, haptoglobin; POD, post-operative day; MVP, mitral valve repair; M-TEER, mitral transcatheter edge-to-edge repair.

**Figure 3:**
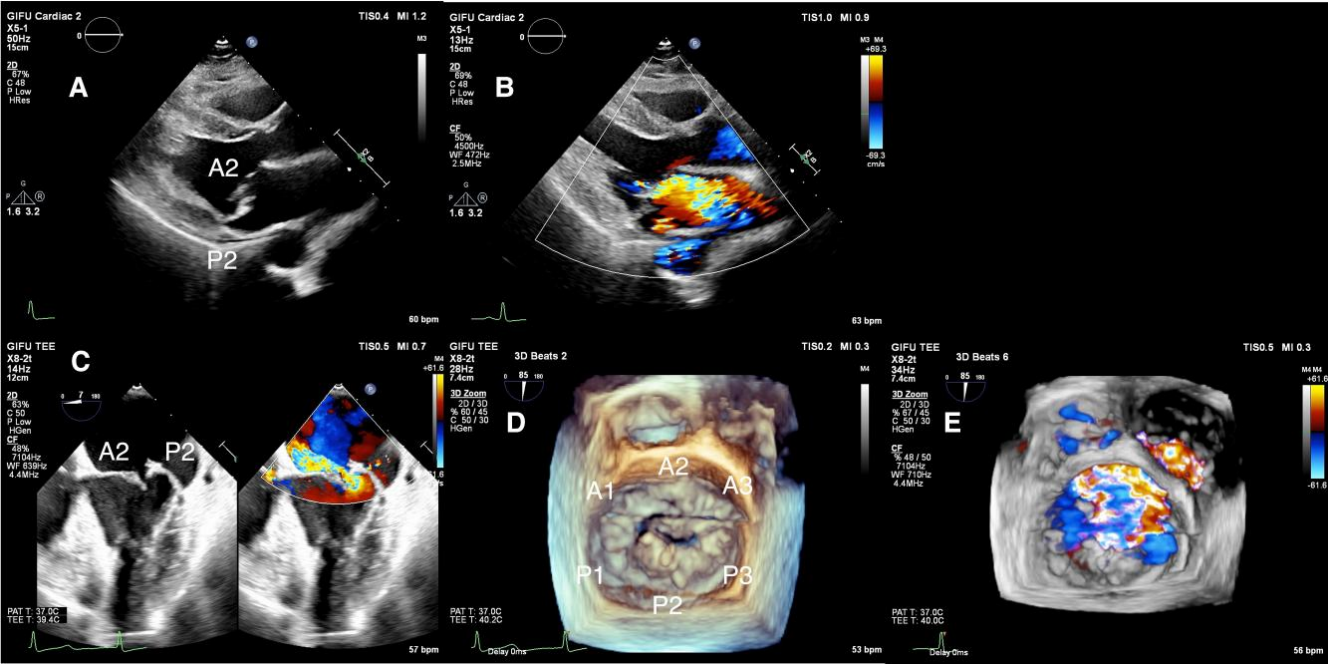
Echocardiography before mitral valve transcatheter edge-to-edge repair (M-TEER). Recurrent P2 prolapse and severe mitral regurgitation by parasternal long-axis views (*A*, *B*) and by transoesophageal echocardiography (*C–E*).

On current presentation, examination revealed a Grade 4/6 holosystolic murmur and increased cardiomegaly (cardiothoracic ratio 60%). Laboratory tests showed B-type natriuretic peptide 33.7 pg/mL (reference range: 8–20 pg/mL), haemoglobin 10.8 g/dL (reference range: 12.0–15.0 g/dL), and lactate dehydrogenase 215 U/L (reference range: 135–225 U/L) with normal liver and renal function. Transthoracic echocardiography demonstrated severe recurrent MR (regurgitant volume 81 mL, fraction 54%, effective orifice area 0.45 cm²) with preserved LV function (ejection fraction 74%). Transoesophageal echocardiography confirmed P2 segment prolapse with mild annular dilation (anterior–posterior axis 34 mm, inter-commissure long axis 42 mm) (*Figure 3A–E*, Supplementary material online, *Video S3*).

**Figure 4:**
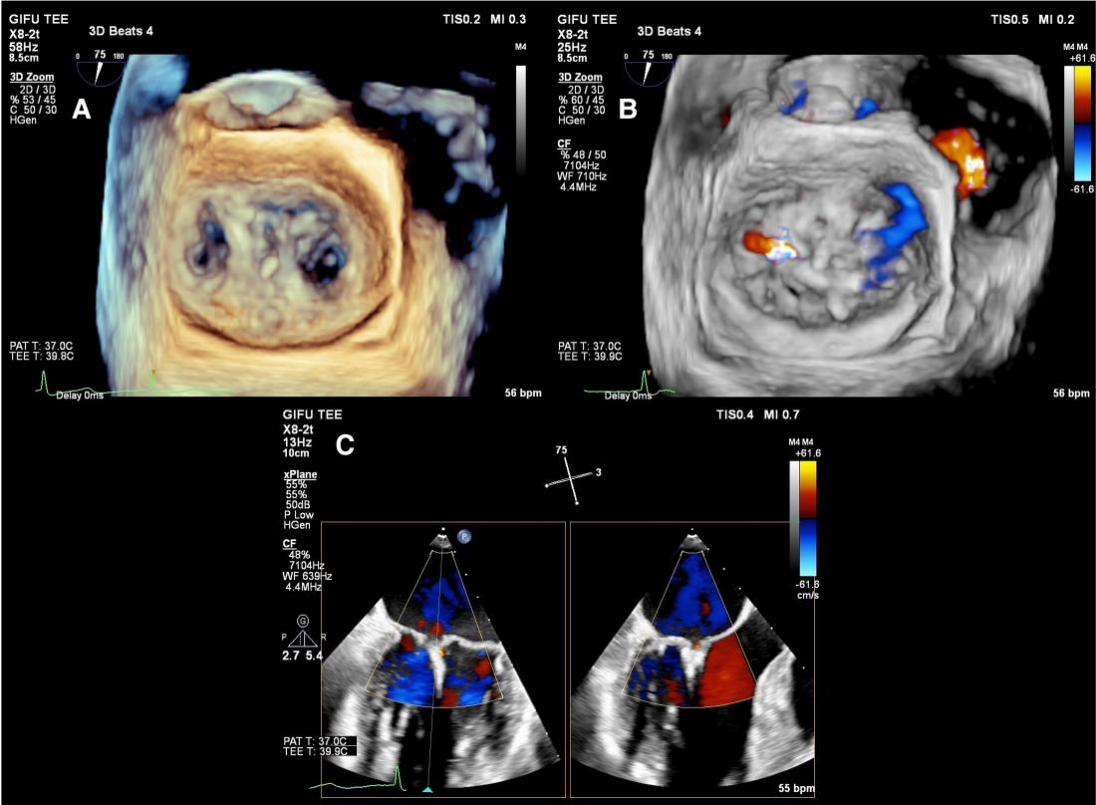
Intraoperative transoesophageal echocardiography during mitral valve transcatheter edge-to-edge repair. (*A*) 3D view: two MitraClips in place. (*B*, *C*) Colour Doppler: residual trivial mitral regurgitation.

Given the risks of redo surgical repair and valve replacement, particularly concerning haemolysis and anticoagulation complications in a patient with HS, the heart team selected M-TEER. To address the patient's underlying HS, the procedure was optimized with minimal catheter manipulation, careful haemodynamic monitoring, and rapid clip deployment to reduce procedural time. Two MitraClip NT devices were placed at the middle segments of the anterior and posterior leaflets (A2/P2) with careful manipulation to reduce mechanical stress on red blood cells, achieving trivial residual MR (*Figure 4A–C*, Supplementary material online, *Video S4*). Post-procedure mitral valve area was 2.2 cm² with a mean gradient of 4 mmHg. Due to the risk of haemolysis, antithrombotic therapy was not initiated. The patient recovered without complications and left the hospital on Day 4.

The patient showed marked functional improvement at the 6-week follow-up. At the 3-year follow-up, although asymptomatic, echocardiography showed progression to moderate MR, with regurgitant jets around and between the clips, but no signs of single leaflet device attachment. At the 5-year follow-up, MR remained moderate with stable mean gradient of 4.0–4.5 mmHg. During follow-up, there were no indicators of haemolysis or progressive anaemia, and the patient maintained her improved functional status (*Table 1*).

## Discussion

This report describes the clinical course of M-TEER for recurrent MR in a patient with HS, with favourable outcomes maintained through 5 years of follow-up. The findings from this case contribute to our understanding of valve intervention strategies in patients with inherited haemolytic disorders.

Hereditary spherocytosis affects ∼1 in 2000 individuals.^1,2^ Abnormalities in membrane proteins—primarily ankyrin, band 3, and spectrins—result in spherical erythrocytes with reduced deformability. Although patients with HS were traditionally considered high-risk candidates for cardiac valve interventions, recent reviews suggest that with modern surgical techniques, haemolysis risk may be lower than previously thought.^3^ However, careful individual risk assessment remains essential given the limited evidence available.

Haemolysis following cardiac valve interventions primarily results from shear stress and direct contact between red blood cells and prosthetic surfaces. The frequency varies significantly by intervention type, with mechanical valves showing higher rates (26–95%) compared to bioprosthetic valves (∼5%).^4^ Mitral valve repair typically demonstrates lower haemolysis rates than replacement, with severe cases requiring reoperation in only about 1% of patients.^3^ Although annuloplasty rings are standard for optimal durability in mitral repairs,^6,7^ we intentionally omitted ring placement given reported cases of ring-associated haemolysis.^8^

Mitral valve transcatheter edge-to-edge repair was initially indicated for high-risk patients with severe degenerative MR and suitable anatomy.^9,10^ Although its application has expanded to post-surgical recurrent MR, technical challenges exist due to ring-related imaging limitations.^5^ Technical success rates in post-ring cases are slightly lower than those reported in native MR (90% vs. 91–96%), and long-term outcomes remain limited in the literature.

When considering management options for recurrent MR in patients with HS, several alternatives exist. Redo surgical repair offers durability but carries increased operative risk and potential for haemolysis. Valve replacement introduces additional prosthetic material that may increase haemolysis risk. The multidisciplinary heart team selected M-TEER based on anatomical suitability, reduced procedural risk, and minimal prosthetic material exposure. Newer devices such as MitraClip XT/XTR and the PASCAL system were unavailable at our institution at the time. Our strategy of using two NT devices addressed the ruptured chordae while avoiding high-velocity residual jets that can cause post-M-TEER haemolysis.^11,12^ In our case, although MR progressed to moderate at 3 years, it remained stable through 5 years without need for reintervention, suggesting the durability of this approach.

While surgical edge-to-edge repair typically requires concomitant ring annuloplasty,^7^ M-TEER has demonstrated beneficial effects on annular geometry even without artificial rings.^13^ This advantage may be particularly relevant for high-risk patients with conditions predisposing to haemolysis, where avoiding additional prosthetic material is desirable.

Our case has several limitations despite favourable outcomes. As a single case report, generalizability is limited. Future research should examine long-term outcomes of M-TEER in larger cohorts of patients with hereditary haemolytic disorders, focusing on haemolysis rates, durability, and optimal patient selection. Specific attention should be paid to technical modifications that might reduce haemolysis risk in this unique population.

This experience demonstrates that M-TEER can be safely and effectively performed in patients with HS and recurrent MR when appropriate technical considerations are implemented. The sustained 5-year outcomes suggest this approach offer a valuable treatment option for patients with hereditary haemolytic disorders, particularly when avoiding additional prosthetic material is desirable. These findings warrant further investigation through larger studies.

## Lead author biography


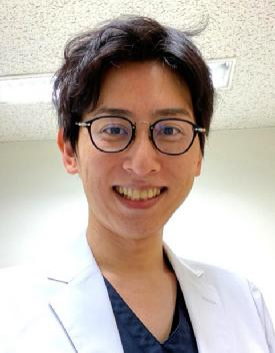


Dr Hiroto Yagasaki is a cardiologist at Gifu Prefectural General Medical Center, Japan, and a visiting researcher at Indiana University School of Medicine, USA. He specializes in echocardiography and cardiac magnetic resonance imaging, focusing on structural heart disease interventions. At his institution in Japan, he leads procedures including transcatheter aortic valve implantation, edge-to-edge valve repair, left atrial appendage closure, and patent foramen ovale closure. He holds board certification in internal medicine, cardiology, echocardiography, and other cardiovascular subspecialties in Japan.

## Supplementary Material

ytaf211_Supplementary_Data

## Data Availability

The data underlying this article are available in the article.

## Notes

**Consent:** Written informed consent was obtained from the patient for publication of this case report and accompanying images, in compliance with COPE guidelines.

**Funding:** This research received no specific grant from public, commercial, or not-for-profit funding agencies.
